# The centrosomal deubiquitylase USP21 regulates Gli1 transcriptional activity and stability

**DOI:** 10.1242/jcs.188516

**Published:** 2016-11-01

**Authors:** Claire Heride, Daniel J. Rigden, Erithelgi Bertsoulaki, Danilo Cucchi, Enrico De Smaele, Michael J. Clague, Sylvie Urbé

**Affiliations:** 1 Cellular and Molecular Physiology, Institute of Translational Medicine, University of Liverpool, Crown Street, Liverpool L69 3BX, UK; 2 Institute of Integrative Biology, University of Liverpool, Liverpool L69 7ZB, UK; 3 Department of Molecular Medicine, Sapienza University of Rome, 00161 Rome, Italy; 4 Department of Experimental Medicine, Sapienza University of Rome, 00161 Rome, Italy

**Keywords:** Ubiquitin, Hedgehog, Gli1, PKA, Phosphorylation, DUB

## Abstract

USP21 is a centrosome-associated deubiquitylase (DUB) that has been implicated in the formation of primary cilia – crucial organelles for the regulation of the Hedgehog (Hh) signaling pathway in vertebrates. Here, we identify KCTD6 – a cullin-3 E3-ligase substrate adapter that has been previously linked to Hh signaling – as well as Gli1, the key transcription factor responsible for Hh signal amplification, as new interacting partners of USP21. We identify a cryptic structured protein interaction domain in KCTD6, which is predicted to have a similar fold to Smr domains. Importantly, we show that both depletion and overexpression of catalytically active USP21 suppress Gli1-dependent transcription. Gli proteins are negatively regulated through protein kinase A (PKA)-dependent phosphorylation. We provide evidence that USP21 recruits and stabilises Gli1 at the centrosome where it promotes its phosphorylation by PKA. By revealing an intriguing functional pairing between a spatially restricted deubiquitylase and a kinase, our study highlights the centrosome as an important hub for signal coordination.

## Abstract (precis)

**Summary:** We identify a Hedgehog-pathway-associated ubiquitin ligase adapter as a direct interaction partner of the deubiquitylase USP21 and discover a close interplay between USP21 and protein kinase A in regulating Gli1.

## INTRODUCTION

Hedgehog (Hh) signaling plays an important role during development and has also been implicated in diverse malignancies, including basal cell carcinomas, medullo- and glioblastoma, as well as pancreatic, colon and breast carcinomas (Amakye et al., 2013; Takebe et al., 2015). Inhibitors of this pathway are under assessment in the clinic as potential anti-cancer therapeutics (Gonnissen et al., 2015; Takebe et al., 2015). The general architecture of the pathway was initially discovered in *Drosophila* (Nusslein-Volhard and Wieschaus, 1980), but much of the cascade is highly conserved from flies to vertebrates (Briscoe and Therond, 2013; Ingham et al., 2011). In contrast to many other signaling pathways, Hh signaling is constitutively repressed by the interplay of two multi-spanning transmembrane proteins, Patched (PTC in *Drosophila*, and PTCH1 and PTCH2 in mammals) and Smoothened (SMO). In the absence of a signal, Patched proteins inhibit SMO, and the key transcription factors, Ci (Cubitus interruptus) in *Drosophila*, and Gli2 and Gli3 in mammals, are converted to transcriptional repressors. Gli2 and Gli3 are phosphorylated by a series of kinases [protein kinase A (PKA), casein kinase 1 (CK1) and glycogen synthase kinase 3 β (GSK3β)], and in particular, Gli3 undergoes processing (limited proteolysis) in a phosphorylation-, ubiquitylation- and proteasome-dependent manner to a transcriptionally repressive form. Activation of the pathway is triggered by binding of Hh ligands to PTCH1 or PTCH2, which triggers their endocytosis and leads to de-repression of SMO, favouring activation of Ci/Gli proteins. These translocate into the nucleus and activate expression of Hh-responsive genes (Hui and Angers, 2011). In mammalian cells, activation of the cascade is localised at the primary cilium, a specialised microtubule-based surface organelle. Release of SMO inhibition leads to SMO, Gli2 and Gli3 translocation to the cilium, and subsequent translocation of the unprocessed transcriptionally active Gli proteins to the nucleus. One of the transcriptional targets is Gli1, which exclusively functions as a transcriptional activator in the pathway. It is the balance of active and repressive Gli proteins that determines signal strength and outcome.

Post-translational modifications play key roles in this cascade (Chen and Jiang, 2013; Gulino et al., 2012). Reversible ubiquitylation can either promote protein degradation, or alter subcellular localisation or activity of a protein. Multiple E3 ligases are thought to play crucial roles in Hh signaling – Cullin1 with the substrate adapter Slimb (in *Drosophila*) and βTRCP (in mammals), and Cullin3 with the substrate adapter Roadkill/HIB (in *Drosophila*) and SPOP (in mammals) and Itch have all been shown to ubiquitylate and destabilise Gli proteins (Jiang, 2006). Approximately 90 deubiquitylases (DUBs) are encoded in the human genome (Clague et al., 2013; Komander et al., 2009). This family of enzymes has been implicated in the regulation of many canonical signal transduction cascades – e.g. NFκB, Wnt, TGFβ and the Ras–MAPK pathway (Clague et al., 2012a; Hayes et al., 2012; Keusekotten et al., 2013). USP8 has been associated with this cascade in *Drosophila*, where it promotes recycling of SMO (Clague et al., 2012b; Li et al., 2012; Xia et al., 2012). However, the role of DUBs in the mammalian Hh-signaling pathway has only recently been explored (Zhou et al., 2015).

We have previously characterised USP21 as the only centrosome- and microtubule-associated DUB in a comprehensive localisation screen (Urbe et al., 2012). USP21 also associates with basal bodies in ciliated cells, and its depletion inhibits the formation of primary cilia, which play a crucial role in the initiation of Hh signaling in vertebrates. Here, we identify a new binding partner of USP21, the BTB-domain-containing cullin E3-ligase adapter protein KCTD6 (KCASH3), which has previously been implicated in Hh signaling (De Smaele et al., 2011). Importantly, we go on to show that either depletion or overexpression of USP21 suppresses Gli1-dependent transcription in human cells. Our results reveal that USP21 stabilises Gli1 at the centrosome and promotes its phosphorylation by PKA, thus contributing to the intricate compartmentalisation of the Hh-signaling pathway.

## RESULTS

### Identification of new USP21-interacting partners

**Fig. 1.**
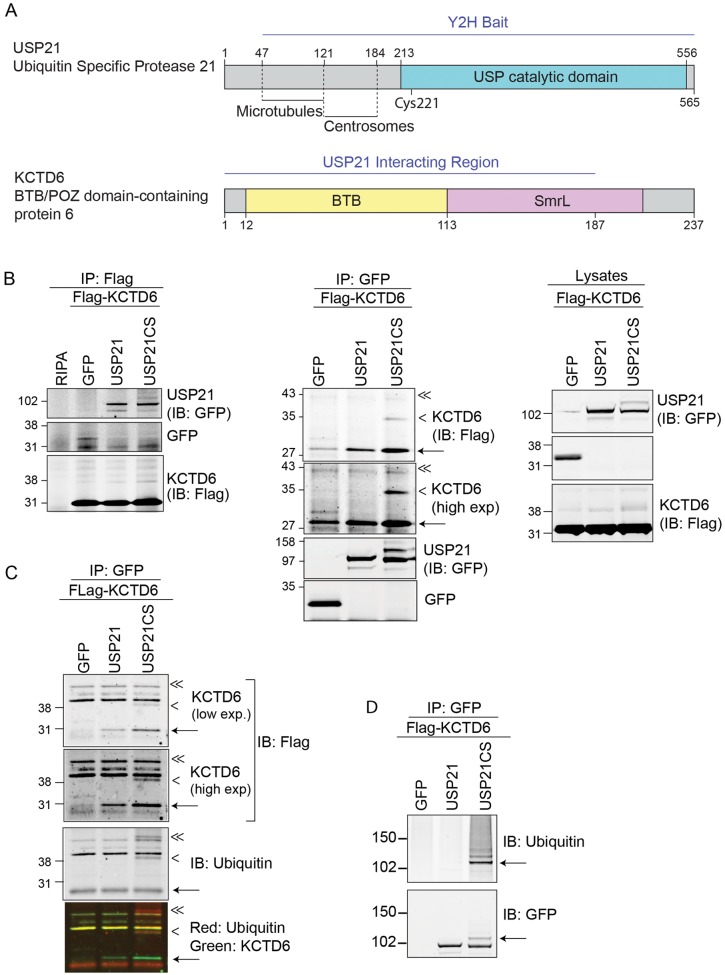
**USP21 interacts with KCTD6, a component of the BTB–CUL3–Rbx1 ubiquitin E3-ligase complex.** (A) A Y2H screen of a normalised human cDNA library using USP21[Δ1–47] as the bait identified the CUL3-substrate-adapter protein KCTD6 (amino acids 1–187). USP, ubiquitin-specific protease; BTB, broad-complex, Tramtrack and Bric-a-brac. An Smr-like domain (SmrL) was identified by *ab initio* modelling (see Fig. 3). (B) Validation of the interaction of USP21 with KCTD6 in human cells. HEK293T cells were transfected with FLAG–KCTD6 and GFP, GFP–USP21 or GFP–USP21CS. Lysates were subjected to immunoprecipitation (IP) with anti-GFP or anti-FLAG antibodies. IB, immunoblot. (C) Inactive USP21 co-precipitated ubiquitylated KCTD6. Lysates were used for immunoprecipitation with an anti-GFP antibody. Interacting proteins were analysed by western blotting with anti-FLAG and anti-Ubiquitin (P4G7) antibodies. Exp, exposure (B,C). Arrowheads indicate mono- (<) and di- (<<) ubiquitylated species of KCTD6. Arrow indicates unmodified KCTD6. (D) The higher-molecular-mass form of USP21-C221S (arrow) corresponds to a monoubiquitylated species. Lysates from the experiment shown in C were subjected to immunoprecipitation with anti-GFP antibodies and probed in parallel with anti-ubiquitin or anti-GFP antibodies.

We have used a yeast two-hybrid (Y2H) approach to search for directly interacting proteins of USP21 that could act as substrates or substrate adaptors in the context of microtubule- and centrosome-dependent processes. Out of a panel of USP21 fragments, only those encompassing amino acids 1–174 and lacking residues 1–47 passed the suitability test by failing to auto-activate reporter genes (Materials and Methods; data not shown). We selected USP21[Δ1–47], which retains all microtubule-binding and centrosome-localisation determinants (Urbe et al., 2012), as a bait to screen a universal normalised human cDNA Y2H library (Fig. 1A). Seven out of ten isolated diploid colonies contained plasmids with in-frame annotated coding sequences, two of which encode ZNF350 (zinc-finger protein 350). The other proteins identified were potassium channel tetramerization domain containing 6 (KCTD6), WD-repeat-containing protein 47 (WDR47), spectrin-repeat-containing nuclear envelope 1 (SYNE1), membrane protein palmitoylated 1 55 kDa (MPP1) and Ankyrin-repeat-domain-containing protein 32 (ANKRD32) (Table 1, Fig. 1A).

**Table 1.**
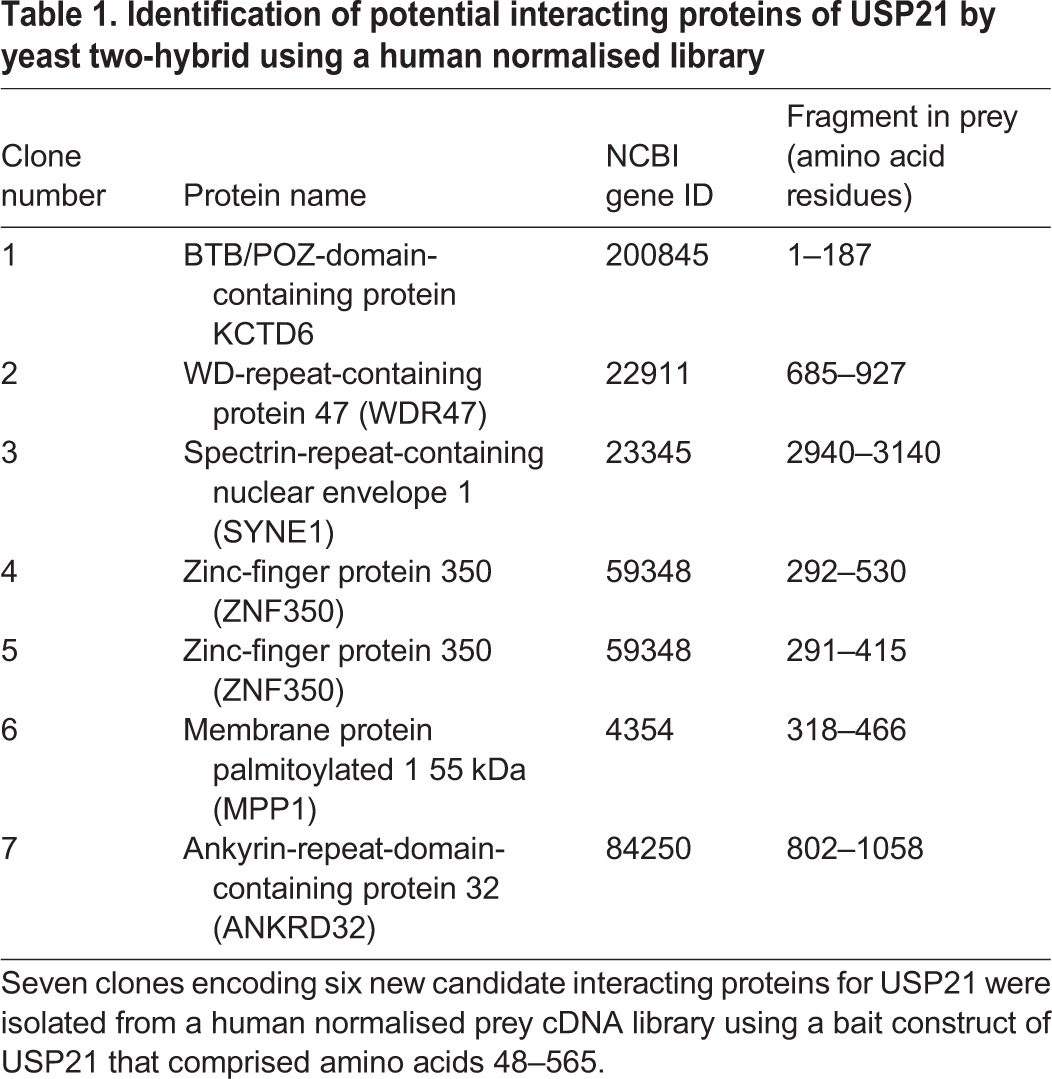
**Identification of potential interacting proteins of USP21 by yeast two-hybrid using a human normalised library**

### USP21 associates with the BTB-domain protein KCTD6

Two of the six new USP21-interacting proteins have been shown to localise to microtubule-based structures. WDR47 colocalises with cytoplasmic microtubules through its interaction with microtubule-associated protein (MAP)8 (Wang et al., 2012a), whereas MPP1 has been localised to the basal body and the ciliary axoneme in murine photoreceptors (Gosens et al., 2007). We were particularly intrigued by KCTD6, a BTB-domain-containing protein that has previously been shown to modulate Hh signaling in association with the related protein KCTD11 (De Smaele et al., 2011). We validated this interaction by co-immunoprecipitating epitope-tagged proteins from HEK293T cells (Fig. 1B). KCTD6 interacts preferentially with catalytically inactive USP21-C221S (USP21CS), which in turn isolates higher-molecular-mass species of mono- and di-ubiquitylated KCTD6 (Fig. 1B,C). USP21CS itself presents as a doublet, a phenomenon commonly observed for catalytically inactive mutants of the USP family – e.g. USP4 and USP15 – which tend to accumulate as a mono- and polyubiquitylated species (Fig. 1D) (Hayes et al., 2012).

### USP21 interacts with CUL3 through the BTB-domain protein KCTD6

**Fig. 2.**
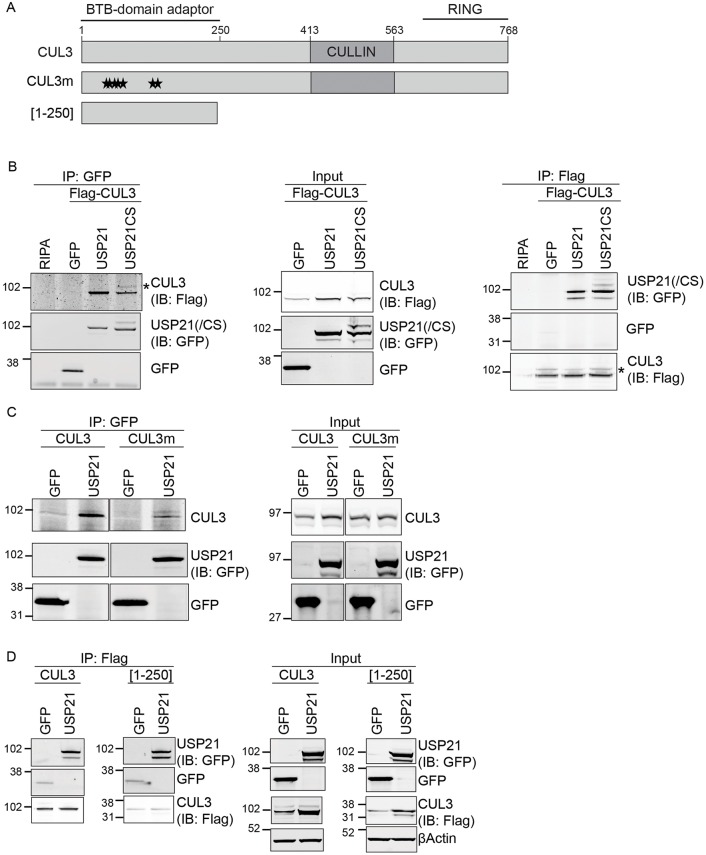
**Indirect interaction of USP21 with cullin-3.** (A) Diagram of the featured CUL3 constructs: full-length CUL3, a BTB-domain-binding mutant (CUL3m) and a dominant-negative C-terminal truncation mutant [CUL3(1–250)]. Asterisks indicate point mutations (see Materials and Methods). (B) USP21 interacts with CUL3 in HEK293T cells. HEK293T cells were transfected as indicated, and lysates were subjected to immunoprecipitation (IP) with anti-GFP and anti-FLAG antibodies as appropriate. Asterisk indicates a higher-molecular-mass form of FLAG–CUL3. (C,D) HEK293T cells were co-transfected with USP21 and with either CUL3m (C) or with CUL3(1-250) (D). Protein lysates were subjected to immunoprecipitation with anti-GFP or anti-FLAG antibodies. The interacting proteins were analysed by western blotting (IB) with anti-GFP and either anti-CUL3 or anti-FLAG antibodies as indicated: USP21(/CS), USP21 and USP21-C221S.

KCTD6 binds to CUL3 through its BTB domain, suggesting that it acts as a classic substrate adapter of a BTB–CUL3–Rbx1 ubiquitin E3 ligase (De Smaele et al., 2011; Lange et al., 2012; Smaldone et al., 2015). We asked whether USP21 interacts with a fully assembled KCTD6–CUL3 ligase complex. Interactions between DUBs and E3 ligases have been established in the literature, but few examples have been analysed in detail (Hayes et al., 2012; Keusekotten et al., 2013; Komander et al., 2009; Schulein-Volk et al., 2014; Sowa et al., 2009; Villeneuve et al., 2013). Both active and inactive USP21 co-immunoprecipitate with CUL3 and vice versa (Fig. 2B; Fig. S1B). Intriguingly, immunoprecipitation of inactive USP21CS enriches a higher-molecular-mass species of CUL3 that could in principle correspond to a neddylated form, which is required for cullin–RING-ligase activation (Bennett et al., 2010). The Nedd8-activating enzyme (NAE) inhibitor MLN4924 did not affect association of FLAG–KCTD6 with endogenous CUL3, nor the amount of USP21 that bound to KCTD6 (Fig. S1A). Importantly, the additional CUL3 band was insensitive to MLN4924, suggesting that USP21CS promotes the stabilisation of ubiquitylated forms of both KCTD6 and CUL3 (Fig. S1B).

The interaction between USP21 and CUL3 is most likely indirect and mediated through KCTD6 given that a functional BTB domain is both required and sufficient (Fig. 2A,C,D). Two closely related KCTD-family members have also been implicated in Hh signaling (Canettieri et al., 2010). The KCTD6-interacting protein KCTD11 (also called KCASH1 or REN), but not KCTD21 (also called KCASH2), bound weakly to wild-type USP21 and strongly to catalytically inactive USP21 (Fig. S1C). However, in contrast to KCTD6, KCTD11 did not appear to be modified in GFP–USP21CS co-immunoprecipitates.

### Identification of a cryptic protein-interaction domain in KCTD6

**Fig. 3.**
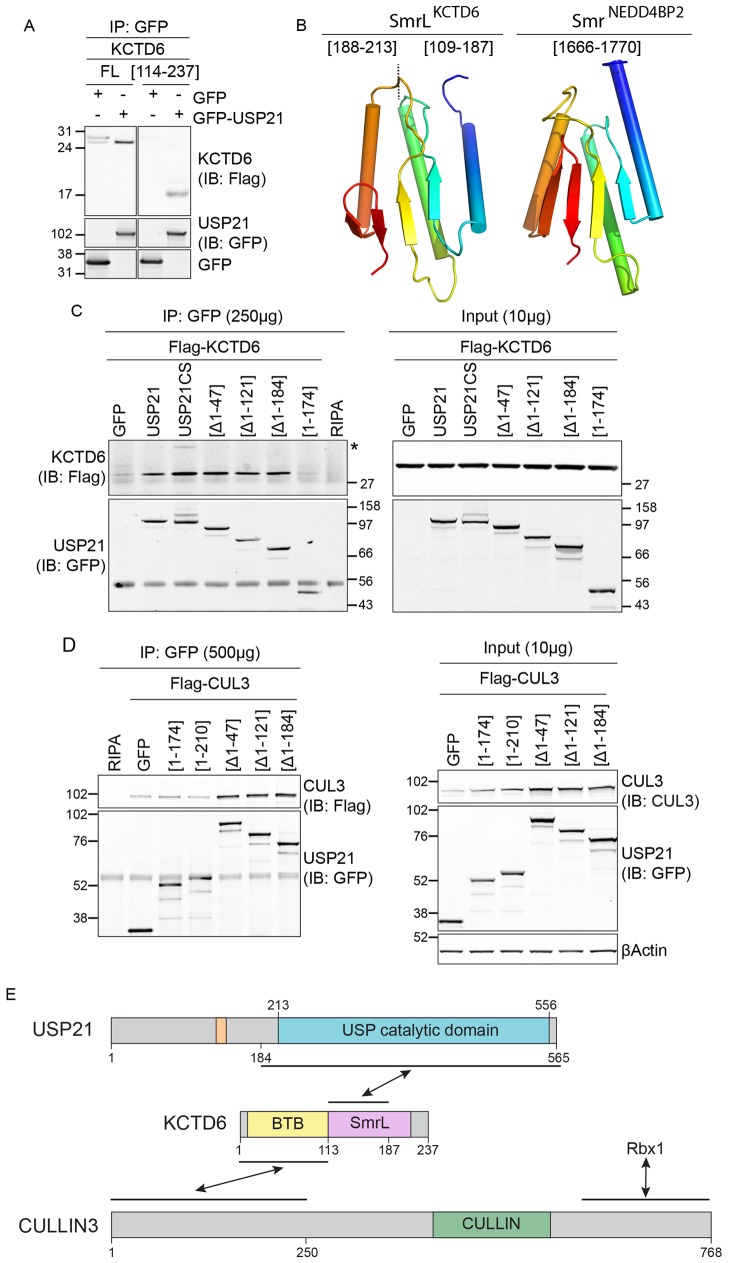
**Mapping the interaction between USP21 and KCTD6–CUL3 E3-ligase complex.** (A) USP21 interacts with KCTD6[114–237]. HEK293T cells were transfected with full-length (FL) or truncated KCTD6, GFP or GFP–USP21, and immunoprecipitations (IPs) were performed and analysed as indicated. IB, immunoblot. (B) The overall fold similarity between the favoured QUARK *ab initio* model of the KCTD6 Smr-like domain [SmrL (109–213)] and the C-terminal Smr domain of human NEDD4BP2 (PDB code 2VKC; Diercks et al., 2008). Each is coloured in a spectrum from blue (N-terminus) to red (C-terminus). A broken line indicates the boundary between the functionally dispensable C-terminal region [188–213] and the remainder. (C,D) HEK293T cells that had been transfected with FLAG–KCTD6 (C) or FLAG–CUL3 (D), and GFP-tagged USP21 fragments were lysed in RIPA buffer. Protein lysates were used for immunoprecipitation with an anti-GFP antibody. Asterisk indicates a monoubiquitylated form of FLAG–KCTD6. (E) Diagram summarising the interactions between USP21, KCTD6 and CUL3, components of the BTB–CUL3–Rbx1 E3-ligase complex. Residues 185–565 in USP21 encompass the necessary elements for the interaction with KCTD6 (residues 114–187). Cullins share a homologous C-terminus that interacts with the RING-finger protein Rbx1.

The best-characterised BTB-domain CUL3 adaptors contain defined substrate interaction domains, such as Kelch, MATH, PHR or zinc fingers (Stogios et al., 2005). No such domains have been described for KCTD proteins, yet the region comprising residues 114–237, which is located directly after its BTB domain, is sufficient for interaction with USP21 (Fig. 3A). The combined results of Y2H and pull-down analyses narrow down the USP21 binding site to the 114–187 region of KCTD6 (Fig. 3E). We hypothesised that this region contains a cryptic protein-interacting domain.

Sensitive bioinformatics tools for detecting distant homologies (HHpred; Soding, 2005) and the Metaserver used to recognise protein folds (Bujnicki et al., 2001) failed to identify recognisable relatives of the KCTD6 C-terminal domain. Nevertheless, the region is rich in predicted secondary structure, suggestive of a well-folded domain. In favourable cases, *ab initio* modelling can accurately predict the overall fold of a queried protein through stitching together fragments of unrelated proteins (Rohl et al., 2004; Xu and Zhang, 2012). Using this approach, the most promising results were obtained for a region comprising residues 109–213, eliminating 24 residues from the C-terminus that some methods indicate might be intrinsically disordered. The favoured model from the QUARK *ab initio* modelling server bears a striking resemblance to the C-terminal Smr domain of human NEDD4-binding protein 2 (NEDD4BP2), with a Z-score of 7.1 for the match, well above the threshold of 2 generally taken as indicating significance [PDB code 2VKC (Diercks et al., 2008); Fig. 3B; Fig. S1D]. According to this model, the C-terminal section (residues 188–213) of this Smr-like (SmrL) domain, containing a helix and a short β-hairpin, is separate from the remainder of the structure, which thus might be able to fold independently. Indeed, separate *ab initio* modelling efforts using a shorter stretch of the peptide, comprising residues 109–187, produced results that included structures similar to those of the modelled SmrL domain (not shown). This might account for the observed association of USP21 with a C-terminally truncated KCTD6 (residues 114–187) in our Y2H screen.

Conversely the Δ1–184 fragment, encompassing the catalytic domain of USP21, is necessary and sufficient to bind to KCTD6 as well as to CUL3 (Fig. 3C,D). Taken together, our data indicate that CUL3, KCTD6 and USP21 can form a multimeric complex in which the catalytic domain of USP21 interacts with the C-terminal SmrL domain of KCTD6, whereas the BTB domain of the latter mediates interaction with CUL3 (Fig. 3E).

### USP21 does not affect KCTD6 and CUL3 protein levels

Based on previously described interactions between DUBs and E3 ligases, we asked whether USP21 might either stabilise or modulate the activity of the KCTD6–CUL3 E3-ligase complex. Neither small interfering (si)RNA-mediated depletion nor overexpression of USP21 altered overall CUL3 protein levels or the relative proportions of active (neddylated) and inactive (non-neddylated) forms (Fig. S2A,B). Unfortunately, none of the commercially available antibodies against KCTD6 that we tested recognised the endogenous protein. FLAG–KCTD6 protein levels were not significantly affected by overexpression of USP21 (Fig. 1B). We conclude that despite the enrichment of ubiquitylated KCTD6 and CUL3 in USP21CS immunoprecipitates, the stability of both proteins is not regulated by USP21. The steady-state protein levels of the sole known substrate of the previously described substrate of the previously described heterodimeric CUL3 E3-ligase substrate adapter complex KCTD6–KCTD11, HDAC1, were also unaffected by depletion or overexpression of USP21 (Fig. S2C,D).

### USP21 regulates the Hh-signaling pathway and modulates Gli1 transcriptional activity

**Fig. 4.**
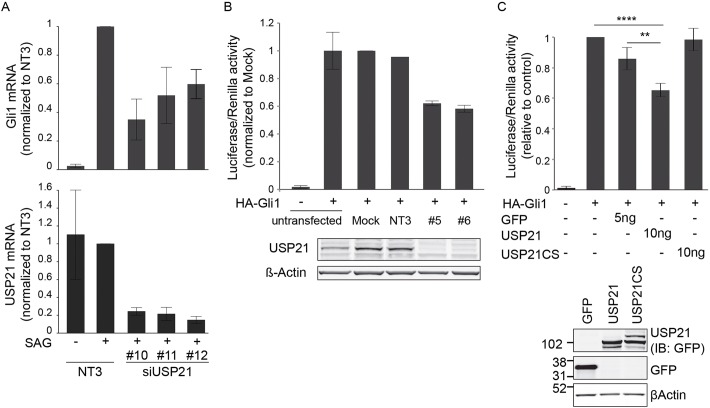
**USP21 modulates Hh signaling and Gli1 transcriptional activity.** (A) NIH3T3 cells were treated with USP21-targeting siRNA oligonucleotides (#10, #11, #12) or a non-targeting control (NT3), serum-starved and then incubated with SAG (100 nM) for 4 h before mRNA extraction. USP21 and Gli1 mRNA levels were measured by performing quantitative reverse-transcription PCR and normalised to RNA levels of Pol2 (*n*=3 independent experiments, mean±s.d.). *x*-axis labels given in bottom panel also apply to the top panel. (B) HEK293T cells were treated with USP21-targeting siRNA oligonucleotides (#5, #6) or non-targeting control oligo (NT3), or were treated with transfection reagent alone (Mock). 24 h before lysis, cells were transfected with Gli-responsive firefly luciferase and *Renilla* luciferase reporters (pGLB3B-12Gli-Luc and pRL-Renilla-TK), as well as with a Gli1 expression construct (HA–Gli1). Efficiency of the knockdown was assessed by western blotting (*n*=2, bars show the range). (C) HEK293T cells were transfected with Gli-responsive firefly luciferase, *Renilla* luciferase reporters, HA–Gli1 and the indicated amounts of GFP, GFP–USP21 or GFP–USP21CS plasmids. Protein expression was assessed by western blotting (IB, immunoblot) for GFP. (GFP, *n*=5; USP21 and USP21CS, *n*=6; mean±s.d.; one-way ANOVA and Dunnett's test, **P<0.01, ****P<0.0001).

KCTD6 has been proposed to act as a negative regulator of the Hh-signaling pathway (De Smaele et al., 2011). We have previously shown that USP21 depletion inhibits the formation of the primary cilium, which also plays a key role in Hh pathway activation in non-transformed cells. In order to assess a potential role of USP21 in Hh signaling, we first measured Gli1 mRNA levels as a transcriptional readout in NIH3T3 cells that had been stimulated with a Smoothened agonist (SAG) (Chen et al., 2002). Depletion of USP21 inhibited SAG-dependent accumulation of endogenous Gli1 mRNA levels (Fig. 4A).

The main transcriptional activator in the pathway is Gli1 itself, which operates through a positive auto-regulatory feedback loop (Hui and Angers, 2011). KCTD6, in concert with KCTD11, has previously been shown to target HDAC1 for ubiquitin-dependent degradation, thereby indirectly modulating the acetylation status and thus transcriptional activity of Gli1. With that in mind, we wanted to assess the ability of USP21 to affect Gli1 transcriptional activity in the absence of extrinsic pathway activation using a Gli1-dependent dual luciferase reporter gene assay. We opted for a heterologous co-expression system of hemagglutinin-tagged Gli1 (HA–Gli1) and GFP–USP21 in HEK293T cells, which permit high levels of transfection efficiency. Importantly, this approach introduces constitutively expressed Gli1 together with the Gli1-dependent reporter construct into the cells. This allows us to pinpoint the stage at which USP21 could regulate Hh signaling, downstream of SMO activation – i.e. downstream of the already established role in primary cilium formation. In this assay, siRNA-mediated depletion of USP21 decreased Gli1 transcriptional activity by ∼40% (Fig. 4B). Likewise, overexpression of GFP–USP21 inhibited Gli1 transcriptional activity to a similar degree, and this inhibition depended on its catalytic activity (Fig. 4C). Altogether, these data indicate that USP21 regulates the Hh-signaling pathway, and either directly or indirectly modulates Gli1 transcriptional activity.

### USP21 interacts with and stabilises Gli1

**Fig. 5.**
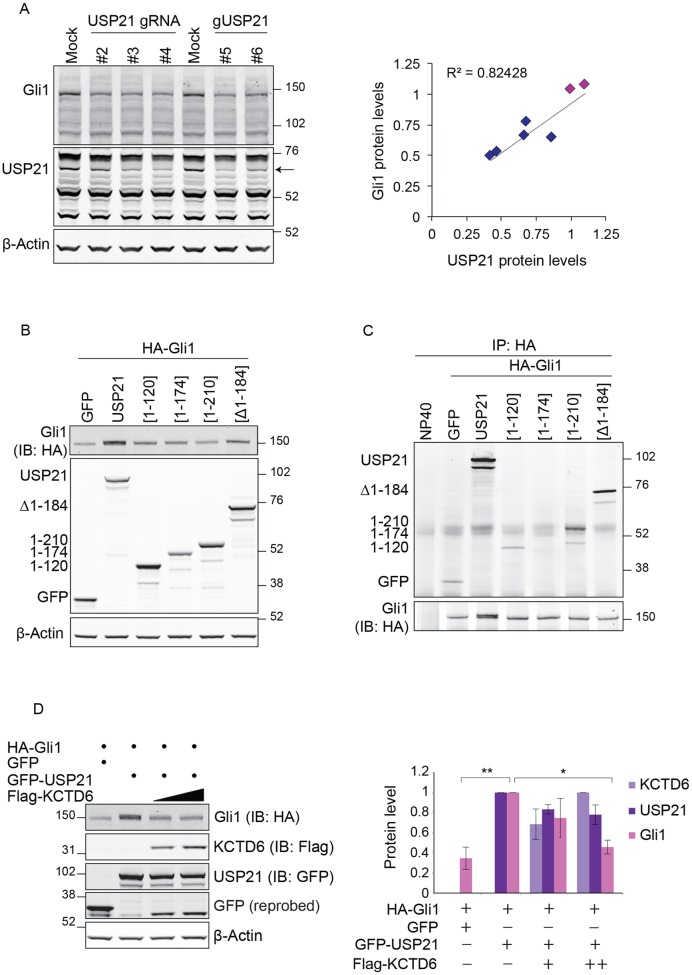
**USP21 regulates Gli1 expression levels.** (A) USP21 knockout results in a decrease in endogenous Gli1 protein. HEK293T cells were transfected with plasmids encoding both Cas9–GFP and one of five independent CRISPR gRNAs targeting USP21 (USP21 gRNA or gUSP21). Cells were lysed in RIPA lysis buffer 72 h after transfection. Protein lysates were assessed by western blotting (a representative blot is shown). Arrow indicates the major USP21 isoform (63 kDa) (Okuda et al., 2013). The scatter plot illustrates the close correlation between USP21 and Gli1 protein levels (relative to actin) across conditions. Pink and blue dots indicate Mock and USP21 gRNA, respectively. (B) USP21 overexpression increases Gli1 protein levels. HEK293T cells were co-transfected with the indicated plasmids, where square brackets indicate the USP21 truncated mutants. Protein lysates were evaluated by western blotting (IB, immunoblot). (C) USP21 interacts with Gli1. Lysates shown in B were subjected to immunoprecipitation with an anti-HA antibody. (D) KCTD6 counteracts USP21-dependent Gli1 stabilisation. HEK293T cells were transfected with HA–Gli1 and GFP–USP21 with increasing amounts of FLAG–KCTD6. Protein lysates were assessed by western blotting. (*n*=3; mean±s.d.; one-way ANOVA and Dunnett's test, *P<0.05, **P<0.01).

We asked whether USP21 regulates the stability of Gli1 and turned to CRISPR-CAS9 technology to knockout USP21 in HEK293T cells, which, like many cancer cells, show autonomous expression of Gli1 (Fig. S3A) (Dahmane et al., 1997; Kinzler et al., 1987; Mazza et al., 2013; Nolan-Stevaux et al., 2009; Tariki et al., 2014). Transient transfection with plasmids encoding both CAS9–GFP and five distinct guide (g)RNAs, targeting the second exon encompassing the start codon of all annotated USP21 isoforms, resulted in a clear decrease of Gli1 protein levels by 25–50% (Fig. 5A). Note that in this configuration, residual USP21 could derive from non-transfected cells. Although these results suggest that USP21 stabilises Gli1, the observed decrease in endogenous Gli1 levels could, in principle, reflect transcriptional suppression (see also Fig. 4A).

We next co-expressed HA-tagged Gli1 together with GFP–USP21 and fragments thereof in HEK293T cells. We observed a clear increase in Gli1 protein levels in cells that co-expressed full-length USP21 (Fig. 5B; Fig. S3B). In order to assess a potential interaction between these two proteins, we immunoprecipitated HA–Gli1 and probed for associated GFP–USP21. Full-length GFP–USP21, but not the shortest N-terminal fragments (comprising residues 1–120 or 1–174), associated with Gli1 (Fig. 5C). Both the unstructured N-terminal region (residues 1–210), required for microtubule and centrosome association, and the C-terminal USP21 region lacking residues 1–184 and harbouring the catalytic domain, were capable of binding to Gli1 independently of each other. Likewise, FLAG-tagged Gli1 and Gli2 were able to co-precipitate Myc-tagged USP21 from HEK293T cells (Fig. S3C,D).

Having shown above that the catalytic domain of USP21 also interacts with the KCTD6–CUL3 E3-ligase complex, we wondered whether KCTD6 would be able to influence Gli1 stability in this setting. Co-expression of KCTD6 with USP21 counteracted its ability to stabilise Gli1 (Fig. 5D). This suggests that the direct interaction of KCTD6 with the USP21 catalytic domain disrupts its regulation of Gli1.

### USP21 stabilises a phosphorylated form of Gli1

**Fig. 6.**
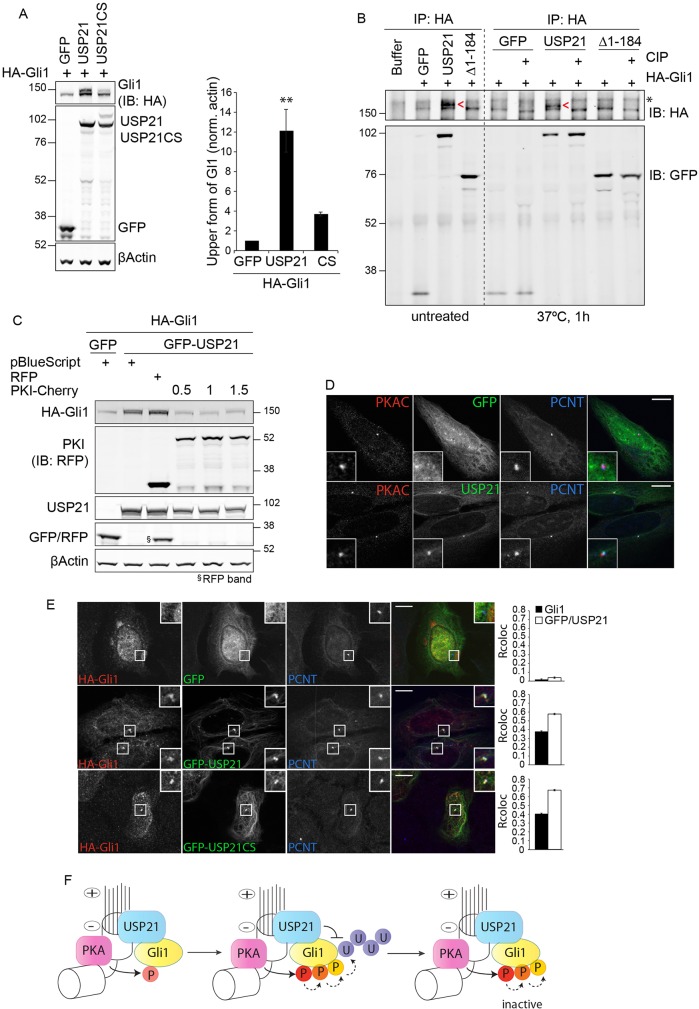
**USP21 recruits Gli1 to the centrosome and promotes its phosphorylation by PKA.** (A) HEK293T cells were transfected with the indicated plasmids for 24 h. Protein expression was assessed by western blotting (IB, immunoblot) for HA and GFP. Graph shows the quantification of the upper form of HA–Gli1 (*n*=3, mean±s.e.m.; one-way ANOVA and Dunnett's test, **P<0.01). (B) HEK293T cells were lysed 24 h after transfection with the indicated plasmids; immunoprecipitated (IP) HA–Gli1 was incubated with alkaline phosphatase (CIP) at 30°C for 1 h. Red arrowheads (<) indicate the upper phosphorylated band. (C) HEK293T cells were transfected with HA–Gli1, GFP or GFP–USP21 and increasing amounts (µg) of PKI–Cherry or RFP control. Cells were lysed 24 h after transfection. Protein levels were assessed by western blotting with anti-HA and anti-RFP antibodies, and membranes were re-probed for GFP. (D) USP21 colocalised with the catalytic subunit of PKA (PKAC, PRKACA) at centrosomes. U2OS cells were transfected with GFP–USP21 or GFP alone as a control. After methanol fixation, cells were stained with anti-PKAC (red) and anti-pericentrin (blue) antibodies. Images were captured with a 3i spinning-disk confocal microscope (63× objective). (E) USP21 and Gli1 colocalize at the centrosome. U2OS cells were co-transfected with HA–Gli1 and GFP–USP21 or GFP–USP21CS. After methanol fixation, cells were stained for Gli1 (red) and pericentrin (blue). Images were captured with a 3i spinning-disk (63× objective). Pearson correlation coefficients for Gli1 or GFP colocalisation with pericentrin were calculated on ≥33 cells per condition (mean±s.e.m.). Scale bars: 10 µm. (F) A working model illustrating the dual role of USP21 in regulating Gli1. USP21 recruits Gli1 at the centrosome and thereby promotes consequential phosphorylation by PKA. PKA could act as a priming kinase for further phosphorylation and ubiquitylation events. Wild-type, but not catalytically inactive USP21, is able to stabilise the labile pool of phosphorylated Gli1. The centrosome and microtubules are also depicted. P, phosphorylation; U, ubiquitin.

The increase of Gli1 protein levels upon USP21 overexpression appears to be at odds with the observed inhibition of Gli1 transcriptional activity in this setting (Fig. 4C). On close inspection, we observed a higher molecular weight form of Gli1 in USP21-overexpressing cells, indicative of a post-translational modification (Fig. 6A). The intensity of this band was substantially reduced in cells that co-expressed the catalytically inactive USP21CS, and the band was absent in cells that expressed truncated versions of USP21 (Fig. S3B). Many components of the Hh-signaling pathway are regulated by acetylation, phosphorylation, ubiquitylation and sumoylation (Chen and Jiang, 2013; Gulino et al., 2012). In particular, Gli transcriptional activity is regulated by several kinases, thus we assessed whether USP21 induces Gli1 phosphorylation. Incubation of immunoprecipitated HA–Gli1 with calf intestinal alkaline phosphatase (CIP) induced loss of the upper band that was only present in cells that overexpressed full-length USP21 (Fig. 6B).

Phosphorylation of Gli transcription factors can either activate or inhibit their transcriptional activity. In the absence of pathway activation, sequential phosphorylation of Gli3, and to a lesser extent of Gli2, by PKA, CKI and GSK3β induces ubiquitin-dependent limited proteolysis to generate a transcriptionally repressive form, GliR (Briscoe and Therond, 2013; Hui and Angers, 2011). Phosphorylation by PKA alone leads to inactivation of Gli2 and Gli3 (Niewiadomski et al., 2014; Tuson et al., 2011), and has been proposed to inhibit nuclear localisation of Gli1 (Sheng et al., 2006). Finally, phosphorylation of Gli1 by ribosomal protein S6 kinase B1 in response to mTOR activation triggers its release from the repressor SuFu, leading to its transcriptional activation (Wang et al., 2012b). Guided by our functional experiments that indicated an inhibitory role of USP21 expression on Gli1-dependent transcription (Fig. 4C), we focused on PKA-dependent phosphorylation as a potential effect of USP21 activity. Co-expression of PKI, a well-established PKA-inhibitor (Scott et al., 1985), abolished the USP21-induced accumulation of phosphorylated Gli1 (Fig. 6C). Strikingly, the decrease of Gli1 phosphorylation was concomitant with a decrease in its protein levels, suggesting that USP21-induced phosphorylation of Gli1 is coupled with its stabilisation (Fig. 6C). Similarly, a pool of Gli2 appeared to be stabilised in a PKI-sensitive manner through co-expression of USP21 (Figs S3C,D and S4A). Altogether, these data indicate that USP21 stabilises inactive Gli1 and promotes its phosphorylation by PKA.

### USP21 promotes Gli1 localisation at the centrosome

How then could the deubiquitylase USP21 induce Gli1 phosphorylation by PKA? We have previously reported that USP21 is most highly enriched at the centrosomes and basal bodies of ciliated cells (Urbe et al., 2012). PKA has previously been shown to be enriched and active at these two locations (Nigg et al., 1985; Terrin et al., 2012; Tuson et al., 2011). U2OS cells allow ready discrimination of intracellular structures owing to their planar geometry and expanded cytoplasm. In these cells, endogenous PKA colocalised with GFP–USP21 at the centrosome (Fig. 6D). Neither wild-type or catalytically inactive USP21 affected PKA protein levels (Fig. S4B). Given that full stabilisation of phosphorylated Gli1 requires the USP21 N-terminal microtubule and centrosome-targeting sequence, we asked whether USP21 influences the localisation of Gli1. Expressed on its own, HA–Gli1 presents with a diffuse, cytoplasmic and nucleoplasmic localisation. However, co-expression of either wild-type or catalytically inactive GFP–USP21 strongly promoted its recruitment to the centrosome (Fig. 6E).

## DISCUSSION

### USP21 interacts with KCTD6, a CUL3 substrate adapter, through its catalytic domain

Our genome-wide Y2H screen yielded six new interacting proteins of USP21. The fact that WDR47 and MPP1 have been shown previously to associate with microtubules or the basal body and axoneme, respectively, makes these likely prospects for future studies (Gosens et al., 2007; Wang et al., 2012a). Here, we have focused exclusively on KCTD6, a CUL3-binding BTB protein that has previously been implicated in the Hh-signaling pathway. This pathway is of particular interest to USP21 function owing to its prominent association with centrosomes, basal bodies, microtubules and primary cilia. We have mapped this interaction to the catalytic domain of USP21 and a new cryptic protein-interaction domain, here called Smr-like (SmrL), that is unique to KCTD6. The Smr domain is generally associated with nuclease function (Fukui et al., 2007). However, the KCTD6 SmrL does not bear the positively charged surface of the NEDD4BP2 Smr structure that is characteristic of DNA- and RNA-binding proteins, nor a suitably positioned catalytic Glu residue (Zhang et al., 2014). Browsing the SCOP structure classification database (http://scop.mrc-lmb.cam.ac.uk/scop; Andreeva et al., 2008), at the level of protein folds that contain Smr domains, reveals a second theme of protein–protein interactions that is exemplified by bacterial sulphurtransferase TusA (Shi et al., 2010) and by the predicted dimeric form of NEDD4BP2 (according to the PISA resource; http://www.ebi.ac.uk/pdbe/pisa; Krissinel and Henrick, 2007). This is clearly consistent with the protein–protein interaction role determined here for the KCTD6 C-terminal domain and is supported by existing precedents of the same fold serving both DNA- and protein-binding functions, e.g. zinc fingers (Matthews and Sunde, 2002). The newly identified SmrL domain is restricted to KCTD6, although KCTD11 and KCTD21 could harbour their own distinct cryptic domains adjacent to the BTB domain. KCTD11, but not KCTD21, has been shown to heterodimerise with KCTD6, providing a possible explanation for its interaction with USP21 in pull-down experiments. USP21, unlike many other members of the USP family, does not contain insertions in its catalytic domain that could encode additional protein interaction motifs or domains. Thus, KCTD6 is the first protein, besides ubiquitin [and ISG15 (Ye et al., 2011)], to be identified as interacting with the catalytic core of USP21, and is a rare example of a core binding protein considering the USP family as a whole.

What then is the role of this interaction? E3-ligase–DUB interactions can protect the ligases from autoubiquitylation [e.g. MDM2 stabilisation by USP7 (Cummins and Vogelstein, 2004), BRAP/IMP stabilisation by USP15 (Hayes et al., 2012)]. Alternatively, the interactions might limit the levels of the respective DUBs or exert fine control over a common substrate. We have not found evidence that USP21 and KCTD6 have reciprocal effects on the stability of the other, nor does USP21 affect global CUL3 stability or neddylation status. However, the fact that co-expression of catalytically inactive USP21 results in the accumulation of minor mono- and di-ubiquitylated species of KCTD6 and CUL3 indicates the potential for USP21 to modulate their ubiquitylation status. Alternatively, USP21 could be acting directly to deubiquitylate the substrates of the cullin RING ligase, in an analogous fashion to USP28, which associates with the cullin-1-adapter FBW7 to deubiquitylate a range of substrates, including MYC (Schulein-Volk et al., 2014). In contrast to FBW7, we have very little knowledge of the substrates targeted by KCTD6. The closely related BTB proteins KCTD11 and KCTD21 undergo hetero- and homodimerisation to target HDAC1 for CUL3-dependent ubiquitylation and degradation (Canettieri et al., 2010). Degradation of HDAC1 promotes the accumulation of transcriptionally inactive acetylated Gli1. De Smaele and colleagues have reported that KCTD6, despite lacking a binding site for HDAC1, could dimerise with its paralogue KCTD11 (but not KCTD21) and contribute to HDAC1 ubiquitylation and degradation (De Smaele et al., 2011). Although we cannot exclude that USP21 directly stabilises KCTD6 substrates, we have not observed any global effects on HDAC1 protein levels in response to siRNA-mediated depletion or overexpression of USP21.

### USP21, a new regulator of the Hh-signaling pathway

Our results suggest that endogenous USP21 positively regulates Hh signaling through two independent mechanisms: firstly, we have previously shown that USP21 depletion interferes with the formation of primary cilia, the specialised organelles that host the initiation of the Hh-signaling cascade in untransformed mammalian cells (Goetz and Anderson, 2010; Urbe et al., 2012). Secondly, we now show that depletion of USP21 not only inhibits SAG-induced transcription in ciliated NIH3T3 cells but also directly interferes with Gli1-dependent activation of a Gli-promoter-driven luciferase reporter in Gli1-transfected HEK293T cells. Although HEK293T cells have previously been shown to be capable of generating primary cilia (Gerdes et al., 2007; Lancaster et al., 2011), under the conditions of our assay (48 h in 0.5% fetal bovine serum) only 30% of cells do so. Whereas we cannot exclude that some aspects of exogenous Gli1-activation rely on primary cilium formation, taken together, our data showing (1) an interaction between USP21 and Gli1, (2) the regulation of PKA-dependent phosphorylation of Gli1 by USP21, and (3) the colocalisation of all three proteins at the centrosome in U2OS cells make a strong case for a regulatory role of USP21 that could be independent of cilium formation.

This positions USP21 downstream of SMO and Patched proteins, and highlights its potential as a new drug target to counteract Hh signaling. This could be of particular relevance in tumours that express aberrant levels of Gli1 or that are otherwise activated downstream of Patched proteins and are thus resistant to the SMO inhibitor Vismodegib (Amakye et al., 2013). In this context, it is interesting to note that *USP21* has been identified in a large-scale screen as one of only few genes that has a significantly increased copy number count in breast tumour samples (Kan et al., 2010). The fact that both depletion and overexpression of USP21 interfere with Gli1-dependent transcription suggests that appropriate USP21 protein levels are key to ensuring optimal Hh-signaling outputs.

### A dual role of USP21 in stabilising phosphorylated Gli1 at the centrosome

Intriguingly, overexpression of USP21 represses Gli1-dependent transcription, despite the fact that the total amount of Gli1 is clearly increased. Associated with this, we observed the accumulation of a phosphorylated pool of Gli1 in USP21-overexpressing cells. Although we have not identified the phosphorylation sites in this study, we provide evidence for the kinase involved in this modification. PKA has recently been shown to phosphorylate multiple serine residues that are conserved in all Gli proteins and to suppress Gli activity in the absence of a Hh signal (Niewiadomski et al., 2014). Our results suggest that USP21 overexpression facilitates these phosphorylation events by recruiting Gli1 either directly or indirectly to the centrosome, which harbours active PKA (Terrin et al., 2012). In ciliated cells, USP21 is localised at the centrosome-derived basal body, where PKA is thought to act on Gli proteins travelling to and from the cilium (Tuson et al., 2011). USP21 might thus promote coincidence of substrate and kinase at these locations. Our data suggest that expression levels of USP21 need to be tightly regulated because both its depletion and overexpression can hamper Hh signaling. However, the role of USP21 exceeds that of an adapter, given that both localisation determinants and catalytic activity are required for maximal stabilisation of phosphorylated Gli1 (Fig. 5B; Fig. S3A). It is conceivable that USP21 regulates both the phosphorylation status and the stability of Gli1 (and possibly Gli2) at the centrosome (Fig. 6F). Our finding provides a unique illustration of the close interplay of a DUB and a kinase at this location, and supports the concept of centrosomes functioning as intracellular signaling hubs or scaffolds (Arquint et al., 2014).

A recent report has identified USP7 as a DUB that is able to stabilise Gli-proteins, by opposing several Ubiquitin–E3-ligase complexes [Cullin1 (Slimb/βTRCP), Cullin3 (HIB/SPOP) and the HECT E3-ligase ITCH] that are involved in Gli protein processing or degradation in the absence and presence of Hh, respectively (Zhou et al., 2015). Our results suggest a more complex scenario in which the restricted localisation of USP21 facilitates the accumulation of an inactive pool of Gli1 at the centrosome. Extending this model to the basal body provides a means of orchestrating Gli protein activity upon entry into or exit from the primary cilium.

## MATERIALS AND METHODS

### Yeast two-hybrid screen

The coding sequence for USP21[Δ1-47] was subcloned from pCR4-TOPO-USP21[Δ1-47] into the pGBKT7 vector (Clontech). The resultant pGBKT7-USP21[Δ1-47] plasmid was sequence verified and transformed into Y2HGold yeast cells. Single colonies that failed to autonomously activate the reporter genes in the absence of prey proteins on tryptophan-lacking X-α-Gal plates, tryptophan-lacking X-α-Gal Aureobasidin-A plates and tryptophan-, adenine- and histidine-lacking X-α-Gal Aureobasidin-A plates were selected for the mating procedure. Briefly, Y2HGold pGBKT7-USP21[Δ1-47] cells were incubated with the human normalised cDNA Y187 prey library (Clontech) in yeast extract-peptone-adenine-dextrose (2×YPAD) medium for 23 h at 30°C. More than 2.4×10^6^ diploid clones were screened and selected for 5 days on synthetic dropout –Trp −Leu X-α-Gal Aureobasidin-A plates. Twenty clones were picked and streaked onto synthetic dropout –Trp –Leu –His −Ade X-α-Gal Aureobasidin-A plates for 7 days. Interacting proteins were identified by yeast colony PCR and subsequently by sequencing of the extracted plasmid.

### Cell culture and transfection

U2OS, HEK293T and NIH3T3 cells were cultured in Dulbecco's modified Eagle medium (DMEM) with Glutamax (Gibco, Carlsbad, CA). Media were supplemented with 10% FBS, 1% nonessential-amino-acid solution and 1% penicillin-streptomycin. All cell lines were routinely tested for mycoplasma contaminations. Transfection of plasmids into HEK293T and U2OS cells was performed with Genejuice (Novagen). For the dual luciferase reporter assays (Promega), plasmids were transfected with Lipofectamine 2000 (Invitrogen) into HEK293T cells (DMEM, 0.5% FBS). Cells were fixed or lysed 24 h after transfection. For siRNA experiments, cells were treated with non-targeting (NT3) or target-specific siRNA oligonucleotides (Dharmacon On-Target Plus, Thermo Fisher Scientific; Table S1) at 40 nM final concentration, once or twice over a period of 96 h (HEK293T) or 120 h (U2OS). NIH3T3 cells were treated with a 12 nM final concentration, twice over a period of 120 h. All siRNA experiments were performed using RNAiMAX (Invitrogen). For Hh-pathway activation, the NIH3T3 cells were first serum-starved in DMEM with Glutamax for 24 h before addition of 100 nM SAG or DMSO for 4 h.

### Quantitative reverse-transcription PCR

Cells were lysed, and mRNA was extracted using the RNAeasy mini kit (Qiagen). cDNA synthesis was performed using 1 µg RNA with RevertAid H-minus M-MuLV reverse transcriptase (Fermentas) using an oligo-dT primer (Promega). Quantitative real-time reverse-transcription PCR was performed in triplicate using iTaq Universal SYBR Green Supermix and a CFX Connect Real-Time PCR detection machine (Bio-Rad). Primer sequences are listed in Table S2.

### Co-immunoprecipitation

HEK293T cells were transfected with a total of 2.4 µg of DNA per 1×10^6^ cells. At 24 h after transfection, the cells were washed in cold PBS and lysed in RIPA lysis buffer (10 mM Tris-HCl pH 7.5, 150 mM NaCl, 1% NP40, 0.1% SDS, 1% sodium deoxycholate) or in NP40-lysis buffer (0.5% NP40, 25 mM Tris-HCl pH7.5, 100 mM NaCl, 50 mM NaF) supplemented with 10 mM N-ethylmaleimide, mammalian protease inhibitor (Sigma) and phosphatase inhibitor cocktail (Roche). Lysates were incubated with primary antibodies and protein-G– or protein-A–agarose beads (P4691 and P2670, Sigma) or with anti-FLAG M2 Affinity Gel (Sigma, A2220) for 1 h at 4°C. Beads were washed with RIPA or YP-IP buffer (0.1% NP40, 25 mM Tris-HCl pH 7.5 and 150 mM NaCl) 3–5 times and once with 10 mM Tris-HCl pH 7.5. Immunoprecipitated samples and 10 µg of the input were evaluated by western blotting.

### Western blot analysis

Cultured cells were lysed with RIPA or NP40 lysis buffers (see above). Proteins were resolved using SDS-PAGE (Invitrogen NuPage gel 4–12%), transferred to nitrocellulose membrane and probed with primary antibodies over night. For anti-ubiquitin antibodies, membranes were boiled for 30 min in distilled water and then blocked with 0.5% fish skin gelatin in TBST. Otherwise, blocking and antibody solutions contained 5% milk. Visualisation and quantification of western blots were performed using an Odyssey infrared scanner (LI-COR Biosciences, Lincoln, NE).

### Antibodies, plasmids and reagents

Antibodies were as follows: anti-USP21 (Sigma, HPA028397; 1:1000), anti-Gli1 (clone L42B10, Biolabs, 2643S; 1:1000), anti-CUL3 (Bethyl, A301-109A; 1:1000), anti-ubiquitin (clone P4G7, Covance, MMS-258R; 1:1000), anti-HA (clone 16B12, Covance, MMS-101P; 1:1000), Polyclonal anti-FLAG (Sigma, F7425; 1:1000), Monoclonal anti-FLAG (clone M2, Sigma, F3165; 1:1000), anti-GFP and anti-RFP (sheep and rabbit, respectively, gift of Ian Prior, University of Liverpool, Liverpool, UK; 1:1000), anti-β-actin (Abcam, ab6276; 1:5000), anti-pericentrin (Abcam, ab4448; 1:1000), anti-PKAC (BD BioSciences, 610980; 1:1000) antibodies. Anti-HDAC1 antibody was obtained from Santa-Cruz (sc-6298; 1:1000), and anti-MYC antibody from Millipore (clone 4A6, 05-724; 1:1000). MLN4924 was obtained from Millennium Pharmaceuticals.

Cloning primer sequences used in this work are given in Table S3. pCXN2-FLAG-KCTD6, pCMV-3HA-Gli1 (G933D), pGLB3B-12Gli-Luc and pRL-Renilla-TK plasmids have been described previously (Canettieri et al., 2010; De Smaele et al., 2011). *KCTD6* was amplified from a liver cDNA library, inserted into pCR4-TOPO (Invitrogen) and subcloned into pCMV-Tag2B-FLAG vector. The DNA encoding KCTD6[114–237] was amplified from pCR4-TOPO-KCTD6, inserted into pET151 vector and subcloned into pCMV-Tag2B-FLAG vector. pGFP-GW, pGFP-GW-USP21, pGFP-GW-USP21CS, pGFP-C1-USP21[1–174], pGFP-C1-USP21[1–210], pGFP-C1-USP21[Δ1–47], pGFP-C1-USP21[Δ1–121] and pGFP-USP21[Δ1–184] plasmids have been described previously (Urbe et al., 2012). pCDNA4-CUL3-[1-250]-FLAG and pCDNA4-CUL3-H2×5 H5×2 [L52A, E55A, Y58A, R59A, Y62A, Y125A and R128A] encoding Cul3[1-250] and Cul3m, respectively, were kindly provided by Michael Rape (University of California, Berkeley, CA). MYC, MYC–USP21 and MYC–USP21CS plasmids have been described previously (Urbe et al., 2012). pCXN2-FLAG-KCTD11, pCXN2-FLAG-KCTD21 and pCDNA-FLAG-Gli1 have been described previously (Canettieri et al., 2010; De Smaele et al., 2011). Murine Gli2 cDNA was kindly provided by Hiroshi Sasaki (Osaka University, Osaka, Japan) and sub-cloned by means of *BamH*I–*Not*I restriction digestion in a pCDNA3 vector engineered to contain an in-frame 5′ FLAG sequence.

CRISPR plasmids expressing Cas9–GFP from a CMV promoter and selected gRNAs from a U6 promoter were kindly provided by Horizon Discoveries (Cambridge, UK). The gRNAs used were designed to target the second exon of the USP21 gene, encompassing the start codon (Table S4). PKI–Cherry plasmid was kindly provided by Oliver Rocks (Max Delbrück Center for Molecular Medicine, Berlin). Smoothened agonist (SAG, CAS 364590-63-6) was purchased from Calbiochem.

### Dephosphorylation assay

Cells were lysed in NP40 lysis buffer supplemented with 10 mM N-ethylmaleimide, mammalian protease and phosphatase inhibitors. Lysates (500 µg) were incubated with protein-G beads and anti-HA antibody for 1 h at 4°C. Beads were washed three times with YP-IP buffer, and once with 1× CutSmart buffer (NEB). Beads were resuspended in 40 µl CutSmart buffer and 3 µl of CIP (NEB) and incubated for 1 h at 30°C, and the reaction was stopped by adding 5 mM of EDTA and subsequent incubation at 65°C for 30 min. Equivalent reaction volumes were loaded onto a gel and evaluated by western blotting.

### Modelling the C-terminal domain of KCTD6

Possible distant homology of the C-terminal domain of KCTD6 with known domains or structures was assessed using HHpred (https://toolkit.tuebingen.mpg.de/hhpred; Soding, 2005) and the Metaserver (https://genesilico.pl/meta2/; Bujnicki et al., 2001). *Ab initio* modelling was performed with ROSETTA (Rohl et al., 2004) or QUARK (Xu and Zhang, 2012). QUARK returns ten ranked fold predictions derived from clustering of *ab initio* models. Likewise, ten candidate structure predictions were derived from 1000 *ab initio* models using the clustering algorithm of the ROSETTA suite. Structural similarity of predictions to known folds was assessed with DALI (Holm and Sander, 1993), and the classifications of proteins of interest were derived using the SCOP database (Andreeva et al., 2008).

### Immunofluorescence

Cells on coverslips were rinsed once with PBS, fixed at −20°C with methanol, blocked in 10% goat serum, and incubated with primary and secondary antibodies in 5% goat serum (1 h each), all in PBS. Secondary antibody was conjugated to Alexa-Fluor-594 or Alexa-Fluor-355. Immunofluorescence images were taken using a 3i spinning disc microscope (63× oil objective) and a digital camera (Hamatsu, CMOS). The images were then processed using SlideBook software and Photoshop CS4 Version 11.0 (Adobe). Pearson's correlation coefficients were calculated using Coloc2 Plugin on Fiji.

### Statistics

*P*-values are indicated as *P<0.05, **P<0.01, ***P<0.001 and ****P<0.0001 and derived either by two-tailed paired *t*-test or, for multiple comparison analysis, by one-way ANOVA and Dunnett's post-hoc test using GraphPad Prism6.

## References

[JCS188516C1] Amakye D., Jagani Z. and Dorsch M. (2013). Unraveling the therapeutic potential of the Hedgehog pathway in cancer. *Nat. Med.* 19, 1410-1422. 10.1038/nm.3389 24202394

[JCS188516C2] Andreeva A., Howorth D., Chandonia J.-M., Brenner S. E., Hubbard T. J. P., Chothia C. and Murzin A. G. (2008). Data growth and its impact on the SCOP database: new developments. *Nucleic Acids Res.* 36, D419-D425. 10.1093/nar/gkm993 18000004 PMC2238974

[JCS188516C3] Arquint C., Gabryjonczyk A.-M. and Nigg E. A. (2014). Centrosomes as signalling centres. *Philos. Trans. R. Soc. Lond. B Biol. Sci.* 369, 2013.0464. 10.1098/rstb.2013.0464 PMC4113108 25047618

[JCS188516C4] Bennett E. J., Rush J., Gygi S. P. and Harper J. W. (2010). Dynamics of cullin-RING ubiquitin ligase network revealed by systematic quantitative proteomics. *Cell* 143, 951-965. 10.1016/j.cell.2010.11.017 21145461 PMC3008586

[JCS188516C5] Briscoe J. and Thérond P. P. (2013). The mechanisms of Hedgehog signalling and its roles in development and disease. *Nat. Rev. Mol. Cell Biol.* 14, 416-429. 10.1038/nrm3598 23719536

[JCS188516C6] Bujnicki J. M., Elofsson A., Fischer D. and Rychlewski L. (2001). Structure prediction meta server. *Bioinformatics* 17, 750-751. 10.1093/bioinformatics/17.8.750 11524381

[JCS188516C7] Canettieri G., Di Marcotullio L., Greco A., Coni S., Antonucci L., Infante P., Pietrosanti L., De Smaele E., Ferretti E., Miele E. et al. (2010). Histone deacetylase and Cullin3-REN(KCTD11) ubiquitin ligase interplay regulates Hedgehog signalling through Gli acetylation. *Nat. Cell Biol.* 12, 132-142. 10.1038/ncb2013 20081843

[JCS188516C8] Chen Y. and Jiang J. (2013). Decoding the phosphorylation code in Hedgehog signal transduction. *Cell Res.* 23, 186-200. 10.1038/cr.2013.10 23337587 PMC3567827

[JCS188516C9] Chen J. K., Taipale J., Young K. E., Maiti T. and Beachy P. A. (2002). Small molecule modulation of Smoothened activity. *Proc. Natl. Acad. Sci. USA* 99, 14071-14076. 10.1073/pnas.182542899 12391318 PMC137838

[JCS188516C10] Clague M. J., Coulson J. M. and Urbe S. (2012a). Cellular functions of the DUBs. *J. Cell Sci.* 125, 277-286. 10.1242/jcs.090985 22357969

[JCS188516C11] Clague M. J., Liu H. and Urbé S. (2012b). Governance of endocytic trafficking and signaling by reversible ubiquitylation. *Dev. Cell* 23, 457-467. 10.1016/j.devcel.2012.08.011 22975321

[JCS188516C12] Clague M. J., Barsukov I., Coulson J. M., Liu H., Rigden D. J. and Urbe S. (2013). Deubiquitylases from genes to organism. *Physiol. Rev.* 93, 1289-1315. 10.1152/physrev.00002.2013 23899565

[JCS188516C13] Cummins J. M. and Vogelstein B. (2004). HAUSP is required for p53 destabilization. *Cell Cycle* 3, 689-692. 10.4161/cc.3.6.924 15118411

[JCS188516C14] Dahmane N., Lee J., Robins P., Heller P. and Ruiz i Altaba A. (1997). Activation of the transcription factor Gli1 and the Sonic hedgehog signalling pathway in skin tumours. *Nature* 389, 876-881. 10.1038/39918 9349822

[JCS188516C15] De Smaele E., Di Marcotullio L., Moretti M., Pelloni M., Occhione M. A., Infante P., Cucchi D., Greco A., Pietrosanti L., Todorovic J. et al. (2011). Identification and characterization of KCASH2 and KCASH3, 2 novel Cullin3 adaptors suppressing histone deacetylase and Hedgehog activity in medulloblastoma. *Neoplasia* 13, 374-385. 10.1593/neo.101630 21472142 PMC3071086

[JCS188516C16] Diercks T., Ab E., Daniels M. A., de Jong R. N., Besseling R., Kaptein R. and Folkers G. E. (2008). Solution structure and characterization of the DNA-binding activity of the B3BP-Smr domain. *J. Mol. Biol.* 383, 1156-1170. 10.1016/j.jmb.2008.09.005 18804481

[JCS188516C17] Fukui K., Kosaka H., Kuramitsu S. and Masui R. (2007). Nuclease activity of the MutS homologue MutS2 from Thermus thermophilus is confined to the Smr domain. *Nucleic Acids Res.* 35, 850-860. 10.1093/nar/gkl735 17215294 PMC1807967

[JCS188516C18] Gerdes J. M., Liu Y., Zaghloul N. A., Leitch C. C., Lawson S. S., Kato M., Beachy P. A., Beales P. L., DeMartino G. N., Fisher S. et al. (2007). Disruption of the basal body compromises proteasomal function and perturbs intracellular Wnt response. *Nat. Genet.* 39, 1350-1360. 10.1038/ng.2007.12 17906624

[JCS188516C19] Goetz S. C. and Anderson K. V. (2010). The primary cilium: a signalling centre during vertebrate development. *Nat. Rev. Genet.* 11, 331-344. 10.1038/nrg2774 20395968 PMC3121168

[JCS188516C20] Gonnissen A., Isebaert S. and Haustermans K. (2015). Targeting the Hedgehog signaling pathway in cancer: beyond Smoothened. *Oncotarget* 6, 13899-13913. 10.18632/oncotarget.4224 26053182 PMC4546439

[JCS188516C21] Gosens I., van Wijk E., Kersten F. F. J., Krieger E., van der Zwaag B., Marker T., Letteboer S. J. F., Dusseljee S., Peters T., Spierenburg H. A. et al. (2007). MPP1 links the Usher protein network and the Crumbs protein complex in the retina. *Hum. Mol. Genet.* 16, 1993-2003. 10.1093/hmg/ddm147 17584769

[JCS188516C22] Gulino A., Di Marcotullio L., Canettieri G., De Smaele E. and Screpanti I. (2012). Hedgehog/Gli control by ubiquitination/acetylation interplay. *Vitam. Horm.* 88, 211-227. 10.1016/B978-0-12-394622-5.00009-2 22391305

[JCS188516C23] Hayes S. D., Liu H., Macdonald E., Sanderson C. M., Coulson J. M., Clague M. J. and Urbe S. (2012). Direct and indirect control of mitogen-activated protein kinase pathway-associated components, BRAP/IMP E3 ubiquitin ligase and CRAF/RAF1 kinase, by the deubiquitylating enzyme USP15. *J. Biol. Chem.* 287, 43007-43018. 10.1074/jbc.M112.386938 23105109 PMC3522295

[JCS188516C24] Holm L. and Sander C. (1993). Protein structure comparison by alignment of distance matrices. *J. Mol. Biol.* 233, 123-138. 10.1006/jmbi.1993.1489 8377180

[JCS188516C25] Hui C.-C. and Angers S. (2011). Gli proteins in development and disease. *Annu. Rev. Cell Dev. Biol.* 27, 513-537. 10.1146/annurev-cellbio-092910-154048 21801010

[JCS188516C26] Ingham P. W., Nakano Y. and Seger C. (2011). Mechanisms and functions of Hedgehog signalling across the metazoa. *Nat. Rev. Genet.* 12, 393-406. 10.1038/nrg2984 21502959

[JCS188516C27] Jiang J. (2006). Regulation of Hh/Gli signaling by dual ubiquitin pathways. *Cell Cycle* 5, 2457-2463. 10.4161/cc.5.21.3406 17102630

[JCS188516C28] Kan Z., Jaiswal B. S., Stinson J., Janakiraman V., Bhatt D., Stern H. M., Yue P., Haverty P. M., Bourgon R., Zheng J. et al. (2010). Diverse somatic mutation patterns and pathway alterations in human cancers. *Nature* 466, 869-873. 10.1038/nature09208 20668451

[JCS188516C29] Keusekotten K., Elliott P. R., Glockner L., Fiil B. K., Damgaard R. B., Kulathu Y., Wauer T., Hospenthal M. K., Gyrd-Hansen M., Krappmann D. et al. (2013). OTULIN antagonizes LUBAC signaling by specifically hydrolyzing Met1-linked polyubiquitin. *Cell* 153, 1312-1326. 10.1016/j.cell.2013.05.014 23746843 PMC3690481

[JCS188516C30] Kinzler K. W., Bigner S. H., Bigner D. D., Trent J. M., Law M. L., O'Brien S. J., Wong A. J. and Vogelstein B. (1987). Identification of an amplified, highly expressed gene in a human glioma. *Science* 236, 70-73. 10.1126/science.3563490 3563490

[JCS188516C31] Komander D., Clague M. J. and Urbé S. (2009). Breaking the chains: structure and function of the deubiquitinases. *Nat. Rev. Mol. Cell Biol.* 10, 550-563. 10.1038/nrm2731 19626045

[JCS188516C32] Krissinel E. and Henrick K. (2007). Inference of macromolecular assemblies from crystalline state. *J. Mol. Biol.* 372, 774-797. 10.1016/j.jmb.2007.05.022 17681537

[JCS188516C33] Lancaster M. A., Schroth J. and Gleeson J. G. (2011). Subcellular spatial regulation of canonical Wnt signalling at the primary cilium. *Nat. Cell Biol.* 13, 700-707. 10.1038/ncb2259 21602792 PMC3107376

[JCS188516C34] Lange S., Perera S., Teh P. and Chen J. (2012). Obscurin and KCTD6 regulate cullin-dependent small ankyrin-1 (sAnk1.5) protein turnover. *Mol. Biol. Cell* 23, 2490-2504. 10.1091/mbc.E12-01-0052 22573887 PMC3386213

[JCS188516C35] Li S., Chen Y., Shi Q., Yue T., Wang B. and Jiang J. (2012). Hedgehog-regulated ubiquitination controls smoothened trafficking and cell surface expression in Drosophila. *PLoS Biol.* 10, e1001239 10.1371/journal.pbio.1001239 22253574 PMC3254653

[JCS188516C36] Matthews J. M. and Sunde M. (2002). Zinc fingers--folds for many occasions. *IUBMB Life* 54, 351-355. 10.1080/15216540216035 12665246

[JCS188516C37] Mazzà D., Infante P., Colicchia V., Greco A., Alfonsi R., Siler M., Antonucci L., Po A., De Smaele E., Ferretti E. et al. (2013). PCAF ubiquitin ligase activity inhibits Hedgehog/Gli1 signaling in p53-dependent response to genotoxic stress. *Cell Death Differ.* 20, 1688-1697. 10.1038/cdd.2013.120 24013724 PMC3824589

[JCS188516C38] Niewiadomski P., Kong J. H., Ahrends R., Ma Y., Humke E. W., Khan S., Teruel M. N., Novitch B. G. and Rohatgi R. (2014). Gli protein activity is controlled by multisite phosphorylation in vertebrate Hedgehog signaling. *Cell Rep.* 6, 168-181. 10.1016/j.celrep.2013.12.003 24373970 PMC3915062

[JCS188516C39] Nigg E. A., Schäfer G., Hilz H. and Eppenberger H. M. (1985). Cyclic-AMP-dependent protein kinase type II is associated with the Golgi complex and with centrosomes. *Cell* 41, 1039-1051. 10.1016/S0092-8674(85)80084-2 2988780

[JCS188516C40] Nolan-Stevaux O., Lau J., Truitt M. L., Chu G. C., Hebrok M., Fernandez-Zapico M. E. and Hanahan D. (2009). GLI1 is regulated through Smoothened-independent mechanisms in neoplastic pancreatic ducts and mediates PDAC cell survival and transformation. *Genes Dev.* 23, 24-36. 10.1101/gad.1753809 19136624 PMC2632164

[JCS188516C41] Nüsslein-Volhard C. and Wieschaus E. (1980). Mutations affecting segment number and polarity in Drosophila. *Nature* 287, 795-801. 10.1038/287795a0 6776413

[JCS188516C42] Okuda H., Ohdan H., Nakayama M., Koseki H., Nakagawa T. and Ito T. (2013). The USP21 short variant (USP21SV) lacking NES, located mostly in the nucleus in vivo, activates transcription by deubiquitylating ubH2A in vitro. *PLoS ONE* 8, e79813 10.1371/journal.pone.0079813 24278184 PMC3838379

[JCS188516C43] Rohl C. A., Strauss C. E. M., Misura K. M. S. and Baker D. (2004). Protein structure prediction using Rosetta. *Methods Enzymol.* 383, 66-93. 10.1016/S0076-6879(04)83004-0 15063647

[JCS188516C44] Schülein-Völk C., Wolf E., Zhu J., Xu W., Taranets L., Hellmann A., Jänicke L. A., Diefenbacher M. E., Behrens A., Eilers M. et al. (2014). Dual regulation of Fbw7 function and oncogenic transformation by Usp28. *Cell Rep* 9, 1099-1109. 10.1016/j.celrep.2014.09.057 25437563

[JCS188516C45] Scott J. D., Fischer E. H., Demaille J. G. and Krebs E. G. (1985). Identification of an inhibitory region of the heat-stable protein inhibitor of the cAMP-dependent protein kinase. *Proc. Natl. Acad. Sci. USA* 82, 4379-4383. 10.1073/pnas.82.13.4379 2989819 PMC390417

[JCS188516C46] Sheng T., Chi S., Zhang X. and Xie J. (2006). Regulation of Gli1 localization by the cAMP/protein kinase A signaling axis through a site near the nuclear localization signal. *J. Biol. Chem.* 281, 9-12. 10.1074/jbc.C500300200 16293631

[JCS188516C47] Shi R., Proteau A., Villarroya M., Moukadiri I., Zhang L., Trempe J.-F., Matte A., Armengod M. E. and Cygler M. (2010). Structural basis for Fe-S cluster assembly and tRNA thiolation mediated by IscS protein-protein interactions. *PLoS Biol.* 8, e1000354 10.1371/journal.pbio.1000354 20404999 PMC2854127

[JCS188516C48] Smaldone G., Pirone L., Balasco N., Di Gaetano S., Pedone E. M. and Vitagliano L. (2015). Cullin 3 recognition is not a universal property among KCTD proteins. *PLoS ONE* 10, e0126808 10.1371/journal.pone.0126808 25974686 PMC4431850

[JCS188516C49] Soding J. (2005). Protein homology detection by HMM-HMM comparison. *Bioinformatics* 21, 951-960. 10.1093/bioinformatics/bti125 15531603

[JCS188516C50] Sowa M. E., Bennett E. J., Gygi S. P. and Harper J. W. (2009). Defining the human deubiquitinating enzyme interaction landscape. *Cell* 138, 389-403. 10.1016/j.cell.2009.04.042 19615732 PMC2716422

[JCS188516C51] Stogios P. J., Downs G. S., Jauhal J. J. S., Nandra S. K. and Privé G. G. (2005). Sequence and structural analysis of BTB domain proteins. *Genome Biol.* 6, R82 10.1186/gb-2005-6-10-r82 16207353 PMC1257465

[JCS188516C52] Takebe N., Miele L., Harris P. J., Jeong W., Bando H., Kahn M., Yang S. X. and Ivy S. P. (2015). Targeting Notch, Hedgehog, and Wnt pathways in cancer stem cells: clinical update. *Nat. Rev. Clin. Oncol.* 12, 445-464. 10.1038/nrclinonc.2015.61 25850553 PMC4520755

[JCS188516C53] Tariki M., Dhanyamraju P. K., Fendrich V., Borggrefe T., Feldmann G. and Lauth M. (2014). The Yes-associated protein controls the cell density regulation of Hedgehog signaling. *Oncogenesis* 3, e112 10.1038/oncsis.2014.27 25111861 PMC5189961

[JCS188516C54] Terrin A., Monterisi S., Stangherlin A., Zoccarato A., Koschinski A., Surdo N. C., Mongillo M., Sawa A., Jordanides N. E., Mountford J. C. et al. (2012). PKA and PDE4D3 anchoring to AKAP9 provides distinct regulation of cAMP signals at the centrosome. *J. Cell Biol.* 198, 607-621. 10.1083/jcb.201201059 22908311 PMC3514031

[JCS188516C55] Tuson M., He M. and Anderson K. V. (2011). Protein kinase A acts at the basal body of the primary cilium to prevent Gli2 activation and ventralization of the mouse neural tube. *Development* 138, 4921-4930. 10.1242/dev.070805 22007132 PMC3201661

[JCS188516C56] Urbé S., Liu H., Hayes S. D., Heride C., Rigden D. J. and Clague M. J. (2012). Systematic survey of deubiquitinase localisation identifies USP21 as a regulator of centrosome and microtubule-associated functions. *Mol. Biol. Cell* 23, 1095-1103. 10.1091/mbc.E11-08-0668 22298430 PMC3302736

[JCS188516C57] Villeneuve N. F., Tian W., Wu T., Sun Z., Lau A., Chapman E., Fang D. and Zhang D. D. (2013). USP15 negatively regulates Nrf2 through deubiquitination of Keap1. *Mol. Cell* 51, 68-79. 10.1016/j.molcel.2013.04.022 23727018 PMC3732832

[JCS188516C58] Wang W., Lundin V. F., Millan I., Zeng A., Chen X., Yang J., Allen E., Chen N., Bach G., Hsu A. et al. (2012a). Nemitin, a novel Map8/Map1s interacting protein with Wd40 repeats. *PLoS ONE* 7, e33094 10.1371/journal.pone.0033094 22523538 PMC3327699

[JCS188516C59] Wang Y., Ding Q., Yen C.-J., Xia W., Izzo J. G., Lang J.-Y., Li C.-W., Hsu J. L., Miller S. A., Wang X. et al. (2012b). The crosstalk of mTOR/S6K1 and Hedgehog pathways. *Cancer Cell* 21, 374-387. 10.1016/j.ccr.2011.12.028 22439934 PMC3350095

[JCS188516C60] Xia R., Jia H., Fan J., Liu Y. and Jia J. (2012). USP8 promotes smoothened signaling by preventing its ubiquitination and changing its subcellular localization. *PLoS Biol.* 10, e1001238 10.1371/journal.pbio.1001238 22253573 PMC3254663

[JCS188516C61] Xu D. and Zhang Y. (2012). Ab initio protein structure assembly using continuous structure fragments and optimized knowledge-based force field. *Proteins* 80, 1715-1735. 10.1002/prot.24065 22411565 PMC3370074

[JCS188516C62] Ye Y., Akutsu M., Reyes-Turcu F., Enchev R. I., Wilkinson K. D. and Komander D. (2011). Polyubiquitin binding and cross-reactivity in the USP domain deubiquitinase USP21. *EMBO Rep.* 12, 350-357. 10.1038/embor.2011.17 21399617 PMC3077245

[JCS188516C63] Zhang H., Xu Q., Lu M., Xu X., Wang Y., Wang L., Zhao Y. and Hua Y. (2014). Structural and functional studies of MutS2 from Deinococcus radiodurans. *DNA Repair* 21, 111-119. 10.1016/j.dnarep.2014.04.012 24811920

[JCS188516C64] Zhou Z., Yao X., Li S., Xiong Y., Dong X., Zhao Y., Jiang J. and Zhang Q. (2015). Deubiquitination of Ci/Gli by Usp7/HAUSP regulates hedgehog signaling. *Dev. Cell* 34, 58-72. 10.1016/j.devcel.2015.05.016 26120032 PMC4627479

